# Commissioning of a gantry‐mounted synchrocyclotron for preclinical FLASH studies utilizing spread‐out Bragg peaks

**DOI:** 10.1002/acm2.70357

**Published:** 2025-11-18

**Authors:** Michael Lowe, Hailei Zhang, Daniel Owen, Arash Darafsheh, Tianyu Zhao

**Affiliations:** ^1^ Department of Radiation Oncology WashU Medicine St. Louis Missouri USA; ^2^ Department of Advanced Development Mevion Medical Systems Littleton Massachusetts USA

**Keywords:** FLASH, proton therapy, synchrocyclotron, ultra‐high dose‐rate

## Abstract

**Background:**

Preclinical studies demonstrate the benefits of ultra‐high dose‐rate (FLASH) radiation, reducing normal tissue toxicity while maintaining tumoricidal effects. Proton FLASH (pFLASH) studies typically use transmission beams, missing the normal tissue‐sparing advantage of the spread‐out Bragg peak (SOBP).

**Purpose:**

This study aims to propose and implement a series of modifications to enable a clinical Mevion S250i gantry‐mounted synchrocyclotron to deliver pFLASH within the SOBP of the proton beam.

**Methods:**

A clinical synchrocyclotron was modified to enable FLASH proton beam delivery using the Mevion FLASH accessory kit, a commercially available tool that allows for the delivery of a single spot SOBP at FLASH dose rates. To ensure accurate beam monitoring, a Faraday cup was utilized to measure the integral charge per delivery at different dose rates and calibrate the FLASH transmission ion chamber (FLASHTic), which is integrated in the FLASH accessory mount attached to the nozzle of the gantry. The FLASHTic was specifically designed to prevent saturation at the dose rates associated with FLASH. To generate the desired single spot SOBP, boron carbide absorbers, a range modulating hole filter, and an 11‐mm‐diameter circle brass aperture were employed on the FLASH nozzle mount.

**Results:**

The results indicate that the FLASHTic measurements demonstrated a strong correlation with the Faraday cup post calibration, suggesting that the FLASHTic can be effectively utilized for both monitoring and terminating the proton beam. The 80%–80% width of the SOBP was 2.01 cm. The peak dose rate at the SOBP proximal peak reached 105.03 Gy/s, with an average of 96.18 Gy/s over five days. Transitioning between FLASH and clinical mode required less than one hour without affecting the clinical beam.

**Conclusions:**

The commissioning of a 230 MeV proton synchrocyclotron for SOBP FLASH delivery was achieved, providing a platform for preclinical small animal studies on pFLASH effects.

## 1 INTRODUCTION

Radiation therapy (RT) at ultra‐high dose rate, also known as FLASH RT, has been the subject of significant research investigations in the past decade due to its potential in improving the therapeutic ratio.^1^, ^2^, ^3^, ^4^, ^5^, ^6^ FLASH effect is referred to an increased differential response between normal and tumor tissues when irradiated at ultra‐high dose rates (≳ 40 Gy/s).^7^ Preclinical studies have shown FLASH effect in different animal models and organs although its underlying mechanism is yet to be fully understood.^8^, ^9^ Taking advantage of full potential of FLASH RT requires a deeper understanding of its radiation biology, which requires robust radiation platforms and dosimetry systems.

The majority of initial published studies on FLASH RT has utilized electron^10^ or photon beams^11^ for irradiation. However, due to the potential of treating deeper seated tumors, there has been a growing emphasis on research and development efforts toward proton RT platforms capable of delivering FLASH dose rates.^12^, ^13^, ^14^ Proton FLASH (pFLASH) irradiation has been demonstrated using isochronous cyclotrons,^15^, ^16^ synchrotrons,^17^ and synchrocyclotrons.^18^, ^19^ These generally are conducted through the modification of a clinical proton therapy beam line.^20^ It is worth noting that most of the published works has been conducted using transmission beams^17^, ^18^, ^21^, ^22^, ^23^, ^24^, ^25^ due to the necessity of delivering the highest available energy to successfully achieve FLASH dose rates. These transmission beams do not fully harness the benefits provided by a proton beam's spread‐out Bragg peak (SOBP) profile, which allows for more precise dose localization and sparing of healthy tissue, but transmission deliveries do provide a simpler method as they do not require any sort of beam modulation.^26^, ^27^ pFLASH irradiation utilizing the SOBP has been demonstrated in several studies using passive beam modifiers.^19^, ^28^, ^29^, ^30^

Isochronous cyclotrons and synchrotrons (within each spill) have quasi‐continuous radiation output, whereas synchrocyclotrons’ radiation output consists of macro pulses that deliver a higher instantaneous dose rate.^31^ Due to the uncertain nature of the radiobiological basis of the FLASH effect, having a variety of platforms to study, this effect is necessary to help discern which beam characteristics (e.g. pulse structure) are important for the FLASH effect.^32^ The current work was built on previous FLASH SOBP feasibility studies,^19^ which was performed in the Mevion factory using an stationary system that provided significant flexibility in optimizing parameters to achieve FLASH dose rates. Considering that feasibility study, the aim of this work is the realization of a clinical proton beam line that could simply and quickly transition to SOBP FLASH delivery mode and to commission the clinical beam for use as a preclinical platform to study FLASH effect in small animals through SOBP beam delivery.

## 2 MATERIALS AND METHODS

### 2.1 Synchrocyclotron FLASH delivery

**FIGURE 1:**
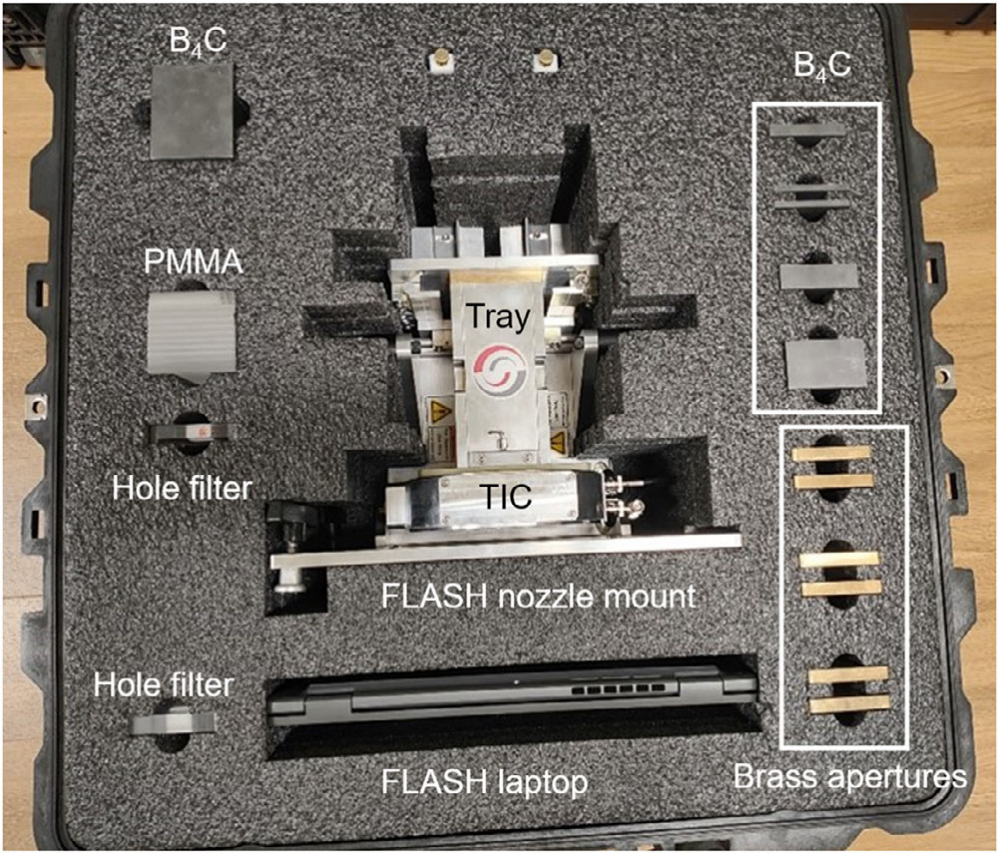
Flash research kit consisted of the FLASH nozzle mount, B_4_C and PMMA absorbers with various thicknesses, hole filter range modulators, brass apertures, and the FLASH laptop.

A clinical proton therapy synchrocyclotron system (HYPERSCAN, Mevion Medical Systems, Littleton, MA) capable of accelerating protons to 230 MeV was modified to support delivery of FLASH proton beams using the Mevion FLASH research kit. The kit consists of the FLASH dosimetry module, FLASH laptop, the nozzle accessory mount equipped with the FLASH transmission ion chamber (FLASHTic), FLASH preamplifier, and beam modulating components that can be inserted into the nozzle accessory mount in variety of configurations depending on the desired treatment depth. The beam modulating components consist of boron carbide (B_4_C) absorbers ranging in water equivalent thickness (WET) from 1 cm up to 16 cm resulting in a cumulative 32 cm of B_4_C, polystyrene sheets with WET values of 1 and 5 mm, brass apertures, and range modulating hole filters capable of producing SOBP in FLASH mode. The FLASH research kit is presented in Figure 1.

**FIGURE 2:**
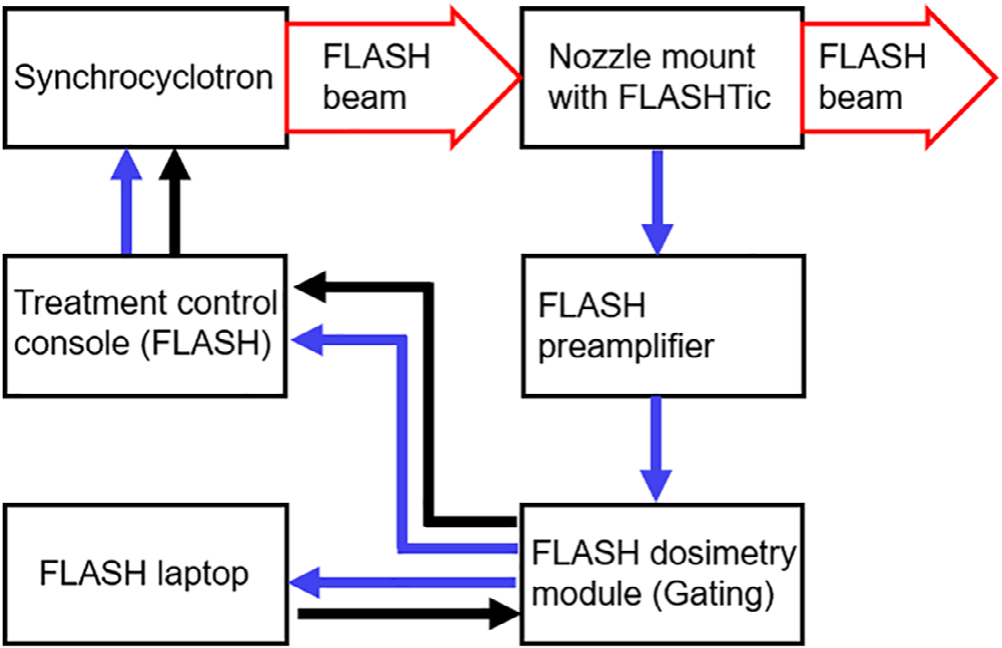
Schematic illustration of the beam pathway from the synchrocyclotron through the nozzle mount, and the signal path and connections between different components for FLASH delivery and active beam monitoring. (Black arrows show the initial signal path starting the radiation. Blue arrows show the feedback path regulating the output.)

Figure 2 schematically illustrates the signal path for FLASH delivery and active beam monitoring. The FLASHTic serves as a specialized transmission ion chamber specifically designed to supplement the clinical monitor chambers within the nozzle. This additional chamber is necessary because the clinical monitor chambers become saturated within the FLASH dose rate range, which causes inaccurate measurement of the charge delivered. By using the FLASHTic, active beam monitoring becomes possible, enabling the beam to be terminated once the desired number of pulses or charge accumulation is achieved. The nozzle accessory mount with the FLASHTic connects to the FLASH dosimetry module via the FLASH preamplifier. Additionally, the FLASH dosimetry module connects to the HYPERSCAN treatment control console and the FLASH laptop, which is responsible for control of the FLASH dosimetry system. Beam delivery is facilitated through service mode on the clinical treatment control console.

After the installation of the hardware, the next step was to tune the beam to achieve the desired FLASH dose rates. This tuning process primarily involved adjusting the charge delivered per pulse. The synchrocyclotron operates at 750 Hz; its radiation output consists of macro‐pulses separated by 1.33 ms temporally. The temporal width of each macro‐pulse can be varied between ∼5–30 µs to change the charge per pulse, hence changing the average dose rate. Typically, for the clinical beam, the charge per pulse is around 8 pC/pulse, which corresponded to a pulse width of 16.5 µs. However, after the tuning process for FLASH delivery, the average charge per pulse increased to approximately 35 pC/pulse, which corresponded to a pulse width of 30 µs. This adjustment allowed for the generation of the desired ultra‐high dose rate (FLASH) beams. Following the installation and tuning of the proton beam, the resulting beam had a full width at half maximum (FWHM) spot size of approximately 4 mm × 5 mm at the full energy of 230 MeV. It should be noted that the adaptive aperture system is not used once the system is set up for FLASH delivery.

### 2.2 FLASHTic calibration

**FIGURE 3:**
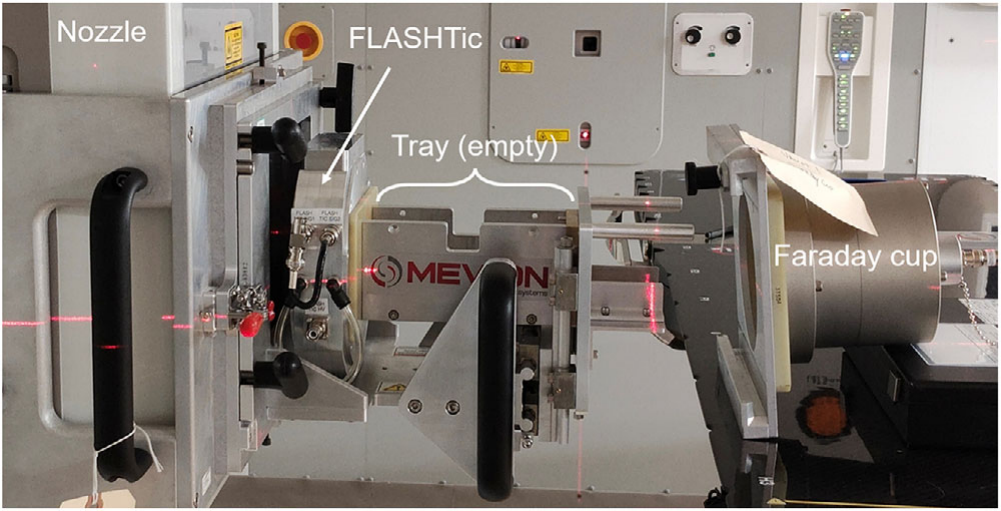
FLASHTic calibration setup with the Faraday cup. The FLASHTic was mounted to the nozzle through the FLASH accessory. The tray for holding beam modulating components is immediately after the FLASHTic. The Faraday cup was placed along the beam beyond the tray.

For each pulse delivered by the synchrocyclotron, the charge accumulated in the FLASHTic is sent as a signal to the FLASH preamplifier, which has two independent charge integrators: (1) the FLASH doseplane integrator that integrates charge synchronously with the pulse frequency and utilizes a background subtraction and (2) the FLASH recycling integrator that integrates continuously without a background subtraction. To ensure an accurate measurement of the charge‐per‐pulse or total charge accumulation, each component is calibrated against a Faraday cup. During the calibration setup, the beam was not collimated, and no B_4_C absorbers were used to modulate the beam energy. The Faraday cup was positioned as close to the end of the FLASH accessory mount as possible, ensuring that the entire beam was incident upon the capacitor, as shown in Figure 3. The calibration procedure is carried out with no beam modulation such that the calibrated charge reading in the FLASHTic matches the effective charge per pulse output from the cyclotron. The calibration process involved comparing the Faraday Cup charge reading to the charge reading for each of the FLASH integrators while varying the charge‐per‐pulse from 1 pC/pulse up to a maximum of approximately 37 pC/pulse, these charge‐per‐pulse values span the lowest and highest achievable dose rates on the Mevion system based on parameters that are adjustable in the clinical setting. The pulse‐by‐pulse charge fluctuation was within 15%. The FLASHTic readouts compared to Faraday Cup measurements were within 2.3% and 0.9% for the doseplane and recycling charge integrators, respectively. These measurements were utilized to generate unique calibration curves for both the FLASH doseplane and recycling charge integrators. Once the calibration curves were derived, a linear fit ($Q_{FC}=0.4879Q_{Rec}+0.0086;R^{2}=1$) was applied between the recycling charge (*Q_Rec_*) integrator and the Faraday Cup charge (*Q_FC_*), and another linear fit ($Q_{FC}=22.798Q_{DP}-0.1887;R^{2}=0.9999$) was derived for the doseplane charge ($Q_{DP}$) integrator. Once the calibration fits were applied to each charge integrator, further measurements covering the previously mentioned charge‐per‐pulse range were again performed without anything in the beamline except for the Faraday cup. These measurements were then used as a validation comparison between the charge integrators on the FLASHTic and the Faraday cup to ensure a strong agreement. This ensured accuracy and linearity of the FLASHTic charge measurements for the usable range of the beam's charge‐per‐pulse output.

### 2.3 Proton FLASH dosimetry

FLASH dosimetry measurements were performed using a calibrated PPC05 (IBA dosimetry) ion chamber due to its thin gap between cathode and anode, which minimizes recombination. These measurements aimed to assess whether the FLASHTic could accurately and repeatably measure and control the dose output to a given target depth such that a given, calibrated dose could be delivered consistently during preclinical animal studies.

**FIGURE 4:**
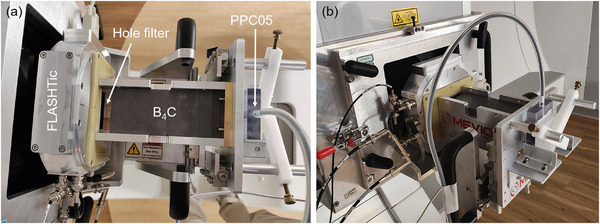
(a) and (b) PPC05 setup for proximal peak dose rate measurements. Boron carbide absorbers were manipulated in this setup to measure the percent depth dose (PDD) curve for the hole filter.

The setup for beam dosimetry, depicted in Figure 4, was designed to measure the dose and dose rate at the proximal peak of the single spot SOBP. A 9.5‐mm‐thick brass aperture with an 11‐mm‐diameter circular opening was placed immediately after the absorber to create a circular field and eliminate stray protons. To determine the location of the proximal peak, a series of depth dose measurements was conducted with a range modulator hole filter in place. This process also facilitated the characterization of the 80%–80% width of the single spot SOBP generated by the hole filter. To ensure accuracy, water tank measurements with the clinical beam were conducted to calculate the WET for each of the B_4_C and polystyrene absorbers and FLASHTic by individually positioning each component into the beam line and analyzing the shift in the depth dose curve.

## 3 RESULTS

### 3.1 Dose‐rate measurement

The average dose rate (ADR) was calculated using Equation (1),^33^

(1) $$\dot{D}=\frac{D}{t}$$

where *t* is the total field delivery time calculated using the pulse time stamps from the FLASH laptop and dosimetry module, and *D* is the absorbed dose derived from IAEA's TRS‐398 protocol.^34^ The ion recombination factor ($P_{ion}$) required special consideration for measurements due to the variation in the charge delivered when using the FLASHTic to gate the beam based on a set number of pulses. To circumvent this issue, $P_{ion}$ measurements were done based on an arbitrarily chosen charge and the FLASHTic gated the beam based on charge to the FLASH Doseplane. This method proved to be considerably more reliable than gating on a specified number of pulses. This method was also used for measurements of $P_{pol}$ for consistency. Two bias voltages of –400 and –150 V were used for the measurements of $P_{ion}$ using the quadratic fitting method.^19^ $P_{pol}$ was calculated with ± 400 V as the bias voltage pairs.

The average ADR, maximum ADR, and minimum ADR at the proximal peak are presented in Table 1. Using the PPC05, dose rates were recorded daily for five consecutive days. The highest proximal peak dose rate measured was 105.03 Gy/s, the lowest dose rate at the proximal peak was 88.7 Gy/s, and a 5‐day average of the ADR was 96.18 Gy/s at the proximal peak of the SOBP.

**TABLE 1:** Average, maximum, and minimum ADRs at proximal peak recorded across all 5 days of measurements. All values are in Gy/s.

	Averaged daily measurements	Max. single measurement	Min. single measurement
Day 1	101.69	105.03	97.30
Day 2	99.09	102.93	96.83
Day 3	96.55	98.62	92.89
Day 4	93.52	97.69	88.70
Day 5	93.60	95.76	89.07
5‐Day average	96.18		

Additional measurements were performed to verify the accuracy of the FLASHTic for beam monitoring and termination. A series of measurements with a varied number of pulses was delivered and used to calculate the necessary charge accumulation on the FLASHTic for a 10 Gy delivery. This was done with Equation (2)

(2)

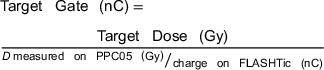

where the TargetGate(nC) is the charge accumulation required to deliver a desired or TargetDose(Gy).

A similar process, using Equation (3), was repeated to assess gating on pulses received by the FLASHTic.

(3)

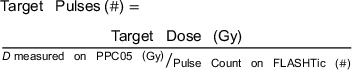




Gating via the accumulated charge method proved to be more reliable for the system with dose variations on average of 0.61%, whereas the pulse count method yielded variations up to 5% with an average difference of 2.7%.

Over the course of the 5‐day period that was used for beam constancy checks, the ADR and charge per pulse in the log files showed a steady decrease. This was monitored intermittently over several months and the ADR stabilized at approximately 83 Gy/s. Working in conjunction with the cyclotron's engineering team, it was determined that the likely cause of this is a combination of thermionic effects affecting the plasma generation and a slight drift in the magnet position that changes the acceleration and extraction efficiency. This is an ongoing area of investigation to find a suitable solution. For preclinical experiments that the stabilized dose rate would not be sufficient, it is suggested that the beam is tuned immediately prior to the experiment to achieve the maximum deliverable dose rate for the synchrocyclotron. The relationship between the decreasing ADR and charge per pulse provided confidence in the calibration of the FLASHTic, which was also verified with Faraday cup measurements intermittently to assess the stability of the calibration. Further measurements over a longer period should provide clarity regarding the re‐calibration frequency required.

### 3.2 Characterization of range modulating hole filter

**FIGURE 5:**
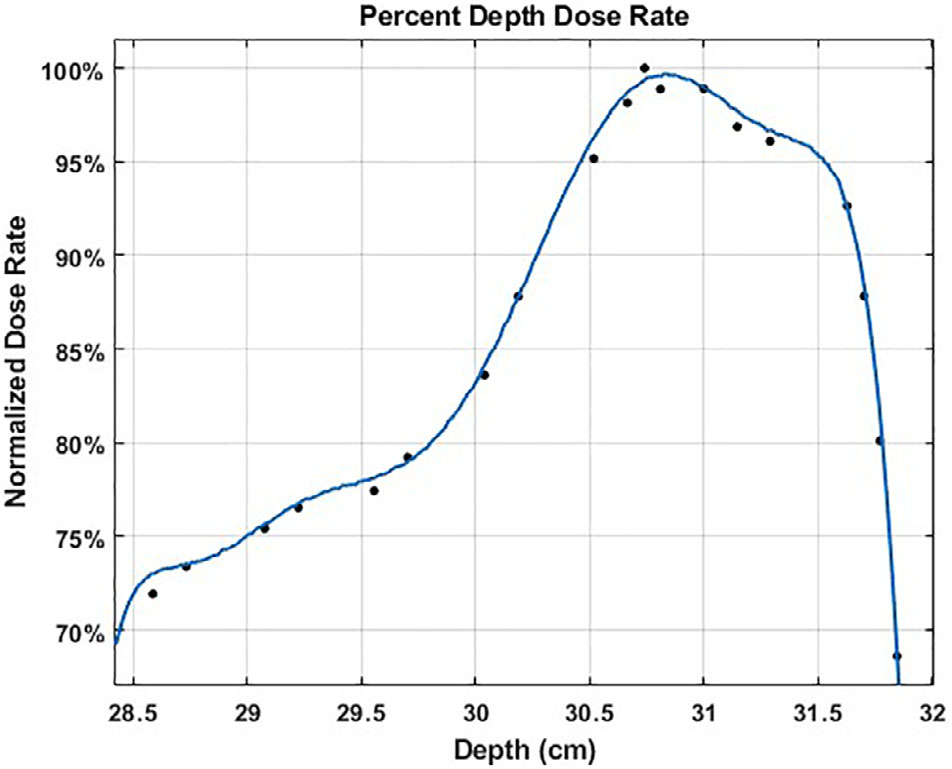
Percent depth dose rate of hole filter measured with B_4_C and polystyrene absorbers yielding an 80%–80% modulation of 2.01 cm. The solid line represents a moving average fit. The un‐normalized data is similar in shape and peaked at 105.03 Gy/s dose rate.

The FLASH nozzle attachment created a physical limitation to using the water tank therefore the hole filter beam modulation and the depth of the proximal peak of the SOBP were measured by sequentially placing in the beamline the blocks of B_4_C and polystyrene absorbers provided in the FLASH kit. The depth varied in sub‐millimeter increments spanning from 28 to 32 cm for the series of measurements used to construct the percent depth dose rate (PDDR) curve for the hole filter. It should be noted that the distal shape of the depth dose profile remains similar in the Mevion system regardless of the initial proton energy due to the absence of an energy selection system.^35^, ^36^ The modulation of the beam by the hole filter, described by Darafsheh et al.,^19^ is shown in Figure 5. The 80%–80% width was measured as 2.01 cm for the single spot SOBP delivery. The average ADR across the 80%–80% SOBP field was calculated to be 91.94% of the dose rate at the proximal peak. During this process the 80%–80% width was chosen to determine what would be considered as the maximum treatment field size for preclinical studies. The 90%–90% width and the 95%–95% width were also measured at this time resulting in beam widths of 1.38 cm and 0.89 cm, respectively. The average ADR across the 90%–90% SOBP was 95.23%, and 97.09% for the 95%–95% SOBP. Due to spatial non‐uniformity of the beam across the PPC05 volume, it has been estimated that approximately 20% under‐measurement of the dose rate would occur, i.e., the dose rate at the center of the beam is expected to be 20% higher than the value reported by the PPC05.^19^

## 4 DISCUSSION

Here, we introduce our preclinical FLASH SOBP proton radiation system and provide insights into the dosimetry associated with the reported dose rates. Furthermore, this study serves the purpose of validating the use of the Mevion FLASH kit, specifically the FLASHTic, as an active beam monitoring tool. The presented system offers a method for measuring the dose and dose rate of individual FLASH beams delivered in preclinical studies, eliminating the need for an additional in vivo dose monitoring system. This feature simplifies the setup and delivery process of FLASH beams for future small animal studies conducted at our facility. By establishing this facility for FLASH delivery, we aim to enhance the understanding and implementation of FLASH radiation techniques in preclinical research. This advancement will contribute to the progress of FLASH‐based therapies by providing a streamlined and efficient method for dose rate determination and active beam monitoring in our experimental settings.

To enhance our understanding of the biological mechanisms underlying the FLASH effect, it is essential that further studies be conducted by various institutions using different delivery platforms. Our system can provide a static FLASH beam that would allow studying the FLASH effect in various organs such as, brain,^37^, ^38^, ^39^ ear,^40^ abdomen,^41^ pelvis,^42^ and skin,^43^ in small animal models as well as in vitro studies. These studies will provide valuable insights into the potential benefits and applications of FLASH radiation therapy. It should be noted that future work and potential hardware adaptations will be required to scale the field size to clinical significance. In line with this aim, our facility has been established with the capability to deliver FLASH dose rates across a SOBP profile. This unique setup allows us to contribute to the technical and biological knowledge required for future clinical development. By conducting research and investigations using this platform, we aim to shed light on the intricacies of the FLASH effect and its potential clinical implications. Collaboration between different institutions and the exploration of various delivery platforms will be crucial in advancing our understanding and harnessing the full potential of FLASH radiotherapy in the future.

## 5 CONCLUSIONS

A clinical proton therapy synchrocyclotron system was successfully demonstrated to simply and efficiently be modified to deliver SOBP pFLASH and transition back to clinical mode without impact on clinical beam. This development allows conducting further preclinical small animal studies to contribute to the radiobiological understanding of the FLASH effect, explore its optimal beam characteristics, and specifically investigating transmission versus SOBP deliveries. Ongoing investigation in collaboration with the accelerator's engineering team aims to improve the performance of the system in the FLASH mode.

## AUTHOR CONTRIBUTIONS

*Conceptualized the study, designed the methodology, and performed the data analysis*: Michael Lowe. *Contributed to data collection, data analysis, and manuscript drafting*: Hailei Zhang. *Assisted with experimental setup and provided technical support throughout*: Daniel Owen. *Ensured accuracy of results and provided critical feedback in drafting and editing the manuscript*: Arash Darasheh. *Supervised the project and contributed to manuscript editing*: Tianyu Zhao. All authors reviewed and approved the final manuscript.

## CONFLICT OF INTEREST STATEMENT

Daniel Owen is a Mevion Medical Systems employee.
